# Role of epithelial sodium channel-related inflammation in human diseases

**DOI:** 10.3389/fimmu.2023.1178410

**Published:** 2023-07-25

**Authors:** Yabin Chen, Xiao Yu, Zhiping Yan, Shuijun Zhang, Jiacheng Zhang, Wenzhi Guo

**Affiliations:** ^1^Department of Hepatobiliary and Pancreatic Surgery, The First Affiliated Hospital of Zhengzhou University, Zhengzhou, China; ^2^National Organ Transplantation (Liver &Kidney Transplantation) Physician Training Centre, Zhengzhou, China; ^3^National Regional Medical Treatment Centre of Henan Organ Transplantation, Zhengzhou, China; ^4^Henan Organ Transplantation Centre, Zhengzhou, China; ^5^Henan Engineering and Research Center for Diagnosis and Treatment of Hepatobiliary and Pancreatic Surgical Diseases, Zhengzhou, China; ^6^Henan Research Centre for Organ Transplantation, Zhengzhou, China; ^7^Henan Key Laboratory for Digestive Organ Transplantation, the First Affiliated Hospital of Zhengzhou University, Zhengzhou, China; ^8^Open and Key Laboratory for Hepatobiliary and Pancreatic Surgery and Digestive Organ Transplantation at Henan Universities, Zhengzhou, China

**Keywords:** epithelial sodium channel, inflammation, hypertension, cardiovascular stiffening, cystic fibrosis, colitis, tumor

## Abstract

The epithelial sodium channel (ENaC) is a heterotrimer and is widely distributed throughout the kidneys, blood vessels, lungs, colons, and many other organs. The basic role of the ENaC is to mediate the entry of Na^+^ into cells; the ENaC also has an important regulatory function in blood pressure, airway surface liquid (ASL), and endothelial cell function. Aldosterone, serum/glucocorticoid kinase 1 (SGK1), shear stress, and posttranslational modifications can regulate the activity of the ENaC; some ion channels also interact with the ENaC. In recent years, it has been found that the ENaC can lead to immune cell activation, endothelial cell dysfunction, aggravated inflammation involved in high salt-induced hypertension, cystic fibrosis, pseudohypoaldosteronism (PHA), and tumors; some inflammatory cytokines have been reported to have a regulatory role on the ENaC. The ENaC hyperfunction mediates the increase of intracellular Na^+^, and the elevated exchange of Na^+^ with Ca^2+^ leads to an intracellular calcium overload, which is an important mechanism for ENaC-related inflammation. Some of the research on the ENaC is controversial or unclear; we therefore reviewed the progress of studies on the role of ENaC-related inflammation in human diseases and their mechanisms.

## 1 Introduction

The epithelial sodium channel (ENaC) is non-voltage-gated and amiloride-sensitive epithelial Na^+^ channel (1). The classic ENaC is composed of α, β, and γ subunits; the α subunit can be replaced by the δ subunit in non-renal tissues (2). The most important function of the ENaC is to maintain the physical and cellular Na^+^ homeostasis by mediating Na^+^ reabsorption in the kidneys, colon, lungs, and skin. ENaC dysfunction is associated with a variety of diseases: ENaC overexpression in the kidneys manifests as increased blood volume and hypertension (3), impaired ENaC in the colon manifests as inflammation of intestinal mucosa and diarrhea (4), and dysfunction of the ENaC in the lungs leads to pneumonia and respiratory distress (5). The expression and activity of the ENaC are regulated by a variety of factors; hormones such as renin-angiotensin-aldosterone system (RAAS), insulin, and vasopressin can maintain Na^+^ metabolic homeostasis by regulating the ENaC. The self-inhibitory effect of Na^+^ on the ENaC is an important piece of negative feedback, as the regulation of the ENaC by mechanical signals is critical in vascular smooth muscle cells (6).

ENaC activation leads to an increased influx of Na^+^, which in turn provides potential energy for cellular material exchange. Increased Na^+^/Ca^2+^ exchanges lead to elevated intracellular Ca^2+^, which activates Ca^2+^-related inflammatory signaling pathways (7). Activated ENaC promotes K^+^ efflux, and increased K^+^ efflux activates NOD-like receptor family pyrin domain containing 3 (NLRP3) inammasome, which can activate immune cells and promote inflammatory cytokine expression (8). Increased intracellular Na^+^ promotes the inward flows of glucose and glutamine, which facilitate tumor growth and migration. Increased intracellular Na^+^ promotes isolevuglandin (IsoLG)-adduct formation and oxidative stress, leading to T-cell activation (9). ENaC-mediated inflammation has an important role in the development of hypertension, vascular sclerosis, pneumonia, cystic fibrosis, nephritis, ulcerative colitis, and tumors. Recently, ENaC activates immune system has been widely reported, while some inflammatory cytokines also have a modulatory effect on the ENaC. In this review, we discuss the roles and mechanisms of ENaC-related inflammation in human diseases.

## 2 The epithelial sodium channel

### 2.1 Structure

The sequences of ENaC subunits and degenerin (DEG) from the nematode *Caenorhabditis* elegans are similar, so they are named the DEG/ENaC family, which also includes the mammalian acid-sensing ion channel (ASIC) (10). Canonical ENaC is a heterotrimer composed of α, β, and γ subunits; the δ subunit can replace the α subunit to form a heterotrimer in non-renal tissues (2). The δβγ channel is less sensitive to proteolytic activation (11) and has a higher IC50 for amiloride than the αβγ channel (12); the δ subunit can also be found in primates and xenopus but is not expressed in mice or rats (13–15). Non-canonical ENaC is composed of two subunits or only homomeric subunits; apart from homomeric γENaC, all non-canonical ENaCs are amiloride sensitive and mediate Na^+^ absorption in oocytes (2). The α, β, γ, and δ subunits are separately encoded by SCNN1A, SCNN1B, SCNN1G, and SCNN1D, respectively (16).

ENaC subunits have two transmembrane domains, intracellular N and C termini, and a large extracellular domain. The ion selectivity filter can specifically discriminate Na^+^; the filter is located in the middle of the transmembrane domains (17). The extracellular domain has protease cleavage sites, which can eliminate the inhibitory effects of the ENaC; it plays an important role in regulating ENaC activation (18, 19). The N-terminal ubiquitylation of the α and γ subunits is related to ENaC endocytosis and degradation (20); both the HGxxR sequence in the N-terminal and the PPPxY sequence in the C-terminal have regulatory effects on the ENaC. Mutations in the HGxxR sequence or the PPPxY sequence leading to abnormal function of ENaC are associated with the occurrence of Liddle syndrome (21) and pseudohypoaldosteronism (PHA) (22).

### 2.2 Distribution

**Figure 1:**
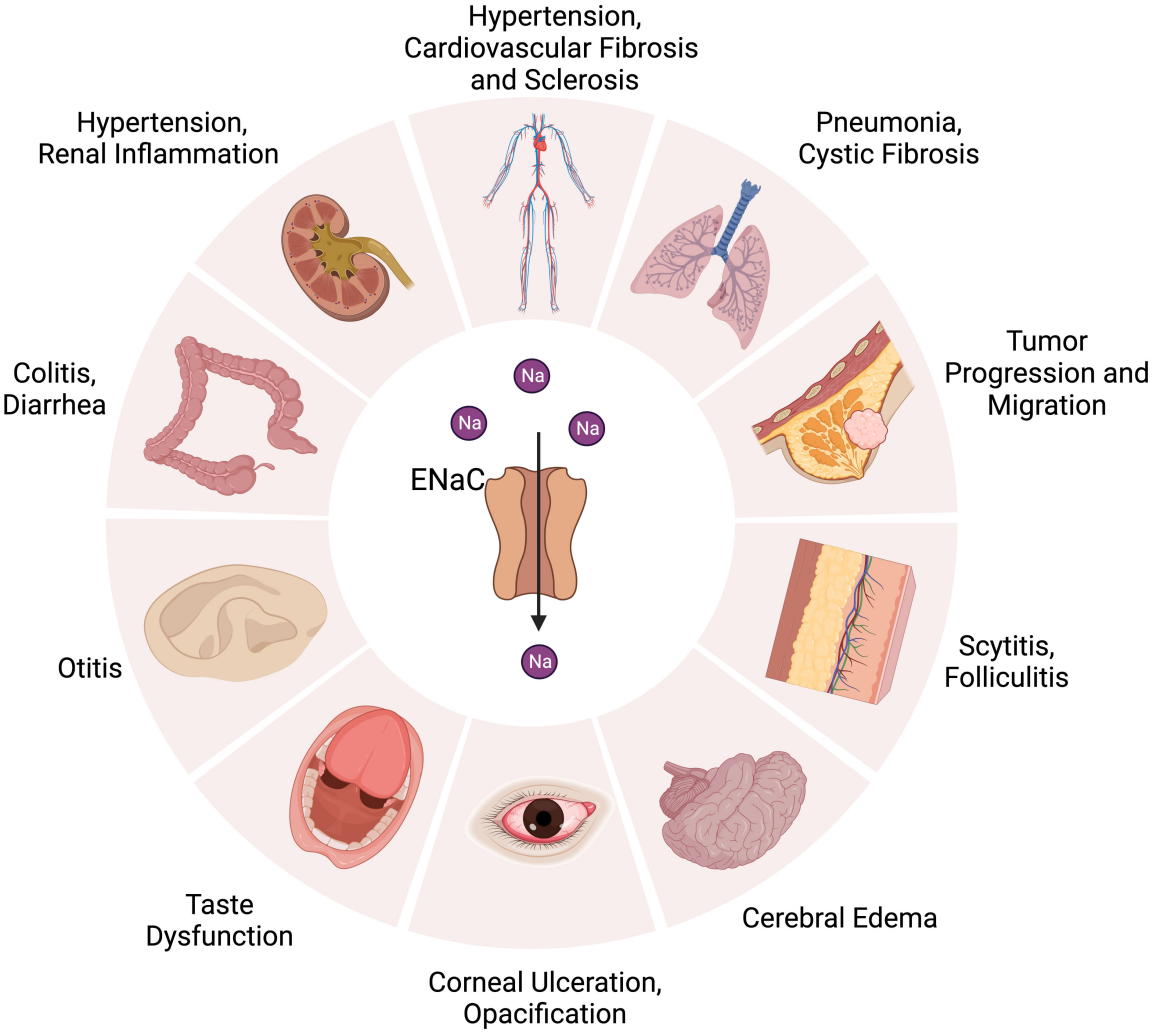
Distribution of ENaC in organs and related diseases. ENaC is associated with hypertension, cardiovascular fibrosis and sclerosis in the cardiovascular system. In the kidney, colon, and skin, ENaC affects water absorption by regulating Na+ transport, which is related to the development of hypertension, diarrhea, renal inflammation, colitis, scytitis and folliculitis. ENaC dysfunction is associated with pneumonia, cystic fibrosis, taste dysfunction, otitis, cerebral edema, corneal ulceration and opacification. ENaC also has an important role in the progression and metastasis of tumor.

The ENaC is firstly found in the apical surface of epithelial cells (23). The αβγ channel is expressed in many organs, such as in the kidneys (distal convoluted tubule, connecting tubule, collecting duct) (24), skin (keratinocyte, sweat gland) (25), vascular system (endothelium, smooth muscle) (26), lungs (alveolar cell, airway cell) (27), colon (28), and tongue (29).The δENaC has also been found in many non-renal organs, such as in the ovaries, brain, liver, lungs, heart, and vessel (13, 16, 30) (**Figure 1**).

### 2.3 Function

The ENaC is critical for epidermal differentiation. Hyperplasia, dehydration, disorder of lipid synthesis and secretion can be found in the αENaC KO mice skin, which leads to death soon after birth (31). Inhibition of the ENaC or blockage of its synthesis can significantly reduce the process of wound healing; the ENaC contributes to wound healing by its activity as a Na^+^ channel and mediator of mechanotransduction (32). The ENaC’s primary role in the colon is the recollection of Na^+^, reducing salt loss in the feces. It is interesting to note that the ENaC is mainly expressed in the distal colon, not in the small intestine or proximal colon (33). A low-salt diet induces increased expression of β and γ subunits but not of α subunits (34). The ENaC controls Na^+^ absorption in the inner ear, which is very important to maintain hearing; dysregulation of the ENaC in the hair cells can lead to hearing loss and vertigo (35). The ENaC can sense mechanical signals in the vascular system such as shear stress (36). The ENaC also has an effect on endothelial stiffness and the release of nitric oxide (NO) (37). The ENaC is critical in infant respiratory epithelium, which can help to remove fluid from the respiratory tract during fetal life; it then plays an important role in maintaining normal airway surface liquid (ASL) (38). Suppression or mutation of the ENaC leads to the development of pulmonary edema or cystic fibrosis (39); surprisingly, symptoms of ENaC overexpression are similar to those of cystic fibrosis (40), although the exact mechanism is not yet clear (**Figure 1**).

### 2.4 Regulation 

**Figure 2:**
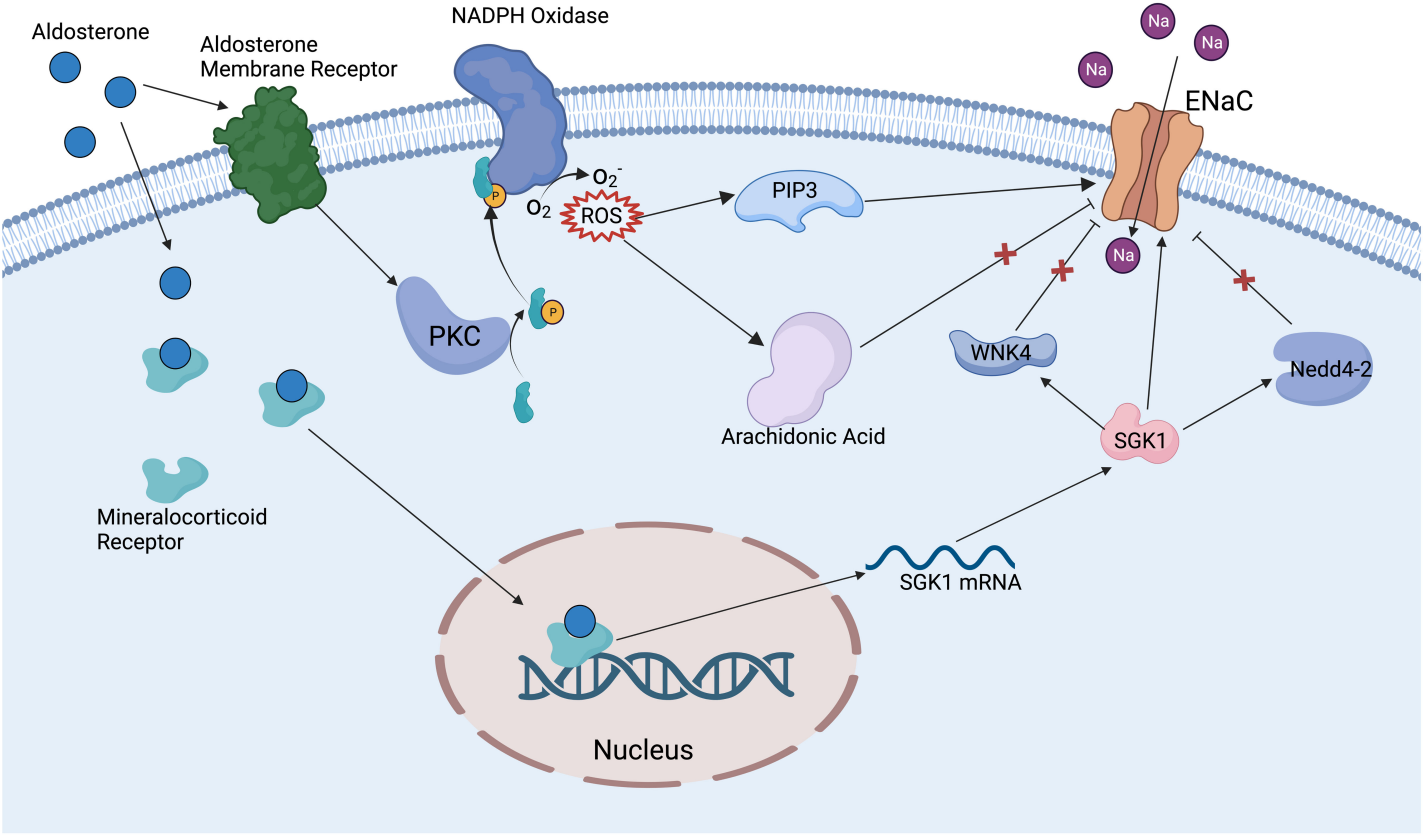
Mechanism of aldosterone regulating ENaC. Aldosterone binds to mineralocorticoid receptors in the cytoplasm and promotes the expression of SGK1. SGK1 can deregulate the inhibition of ENaC by WNK4 and Nedd4-2 ubiquitinates, also can directly activate ENaC. Aldosterone binds to aldosterone receptors on the cell membrane to activate PKC and NADPH oxidase, then promotes the production of ROS. ROS activates ENaC via PIP3 and relieves the inhibition of ENaC by the arachidonic acid metabolic pathway.

There are many factors that can regulate the ENaC, including ions, mechanical signals, hormones, phospholipids, posttranslational modifications, and other proteins. The ENaC can be regulated by extracellular Na^+^ and intracellular Na^+^. An increase of Na^+^ leads to EnaC allosteric change and reduction in channel open probability; this phenomenon is called feedback inhibition (18). Increased extracellular Na^+^ leads to the downregulation of ENaC expression in renal epithelium, whereas in vascular endothelium it leads to increased ENaC expression, and the exact mechanism of the difference in responses is unclear (41). The ENaC provides a mechanosensory function in vascular endothelium and smooth muscle cells; shear stress can activate the ENaC then regulate NO release and vessel vasodilation (42). Hypovolemia or reduced glomerular filtration rate can stimulate the RAAS, further activate the ENaC, and lead to increased Na^+^ reabsorption, which in turn raises blood pressure (43). Aldosterone is one of the main regulators of the ENaC; especially in the distal nephron, aldosterone binds to the mineralocorticoid receptor (44), then the complex translocates into the nucleus and promotes associated gene expression (45) (**Figure 2**). Insulin can reduce internalization and ubiquitylation of ENaC, phosphorylation of insulin receptor activates SGK1 kinase by phosphatidylinositol3-kinase (PI3K), which in turn modulates ENaC activity (45–47). Vasopressin increases the expression of the β and γ subunits, but fewer effects on the α subunit have been proven *in vivo* and *in vitro*; this effect may be achieved by vasopressin receptors (48). Several phospholipids have been reported to have a regulatory effect on the ENaC. Phosphatidylinositol (4, 5)-bisphosphate (PIP2) and phosphatidylinositol (3–5)-triphosphate (PIP3) have a direct positive regulatory role on the C-terminal of β and γENaC (49). Thus, many substances that have a regulatory effect on PIP2 and PIP3 can indirectly modulate ENaC activity, such as phospholipase C (49), myristoylated alanine-rich C-kinase (50), and phospholipase β3 (51).

Posttranslational modifications have an important modulatory effect on the ENaC. Nedd4-2 (neural precursor cell expressed developmentally downregulated protein 4-2) can promote specific lysine residues ubiquitination in the N-terminal regions of α and γENaC, ultimately promoting ENaC endocytosis and degradation (52). Dexamethasone promotes ENaC expression by regulating DNA methylation, which may be a potential therapeutic mechanism for cystic fibrosis (53). Extracellular signal-regulated kinase (Erk) has been reported to phosphorylate PY motifs in βENaC, rapidly modulating ENaC activity (54). Deubiquitylating enzymes (DUBs and USPs) can reduce α and γENaC ubiquitylation and internalization enhance ENaC stability and activity (55). N- and C-terminal regions on β or γENaC can be palmitoylated on specific cysteine residues, which can reduce channel open possibility (this is one of the mechanisms of Na^+^ self-inhibition (56)). Proteases can activate ENaC by cleaving inhibitory fragments; for example, golgi-resident protease furin can release inhibitory fragments in α and γENaC, and prostasin and low-dose trypsin can also activate ENaC through a similar mechanism (57). WNK lysine deficient protein kinase 1 (WNK1) has been proven to activate the ENaC by SGK1 pathway (58), while WNK4 has an inhibitory effect on the ENaC (59) (**Figure 2**). Amiloride hydrochloride is a specific inhibitor of the ENaC, with an IC50 of 1μM, and is widely used in clinical treatments and in the basic research of the ENaC (60).

### 2.5 Effect on other ions and channels

The ENaC’s fundamental function is to regulate the transport of Na^+^; increased intracellular Na^+^ can further influence other ions and channels (41). ENaC mediates the influx of Na^+^, which is pumped out of cells via the Na^+^/K^+^-ATPase (NKA) (61). Reabsorbed Na^+^ can create the driving force to excrete K^+^ through apical secretory K^+^ channels (62, 63); these are important pathways for K^+^ excretion, but the ability of ENaC to directly secrete k^+^ is limited, because ENaC is more (>100-fold) selective to Na^+^ than K^+^ (12). The ENaC has been shown to interact with the HCO_3_^−^/Cl^−^ exchanger and to some extent can affect blood HCO_3_^−^ and Cl^−^ levels (64, 65). The ENaC mediates increased intracellular Na^+^ and facilitates the exchange of Na^+^ with Ca^2+^ through the Na^+^–Ca^2+^ exchanger (NCX), which causes a Ca^2+^ overload and further activates downstream signaling pathways (7, 8). Some hormones or kinases have regulatory effects on multiple ion channels, which may lead to the ENaC indirectly affecting other ions and channels, as both SGK1 and aldosterone have effects on the ion channels of Na^+^, K^+^, Ca^2+^, and Cl^−^ (66, 67). Reports about the effects of the ENaC on other ions and channels are lacking, and therefore need further study.

Pendrin is a Cl^−^/HCO_3_^−^ exchanger that can be seen in the intercalated cells. The function of ENaC is downregulated in pendrin-null kidney (68). In H^+^/K^+^-ATPase type 2 (HKA2)-null mice, the expression of α and γENaC, pendrin are upregulated, but the expression of Na^+^/Cl^−^ cotransporter (NCC) is downregulated (69). The ENaC has a regulatory effect on the NCC, and γENaC knockout leads to impaired excretion of K^+^ and increased NCC activation (70). ZIP2/SLC39A2 is a splice isoform of the Zn^2+^ importer, and when inversely correlated with intracellular Zn^2+^ can induce ENaC expression and activation in cystic fibrosis (71). HKalpha2 is one of the H^+^/K^+^ ATPase subunits in the colon, and decreased ENaC-mediated Na^+^ reabsorption has been observed in HKalpha2 homozygous knockout mice (72). The cystic fibrosis transmembrane conductance regulator (CFTR) can mediate Cl^−^ and HCO_3_^−^ efflux. Na^+^ enters cells through the ENaC and is pumped out by NKA; this generates a transepithelial electrical gradient of Cl^−^, which is absorpted into cells by CFTR, and decreased Cl^−^ in cytosolic increases ENaC expression and Na^+^ reabsorption (73, 74). Na^+^/H^+^ exchanger (NHE3), bumetanide-31 sensitive Na^+^/K^+^-2Cl^−^ transporter (NKCC2), NCC, and the ENaC work together in the kidneys to regulate the reabsorption and secretion of Na^+^ and K^+^ (75).

## 3 Role of epithelial sodium channel-related inflammation

### 3.1 Epithelial sodium channel-related inflammation in cardiovascular system

**Figure 3:**
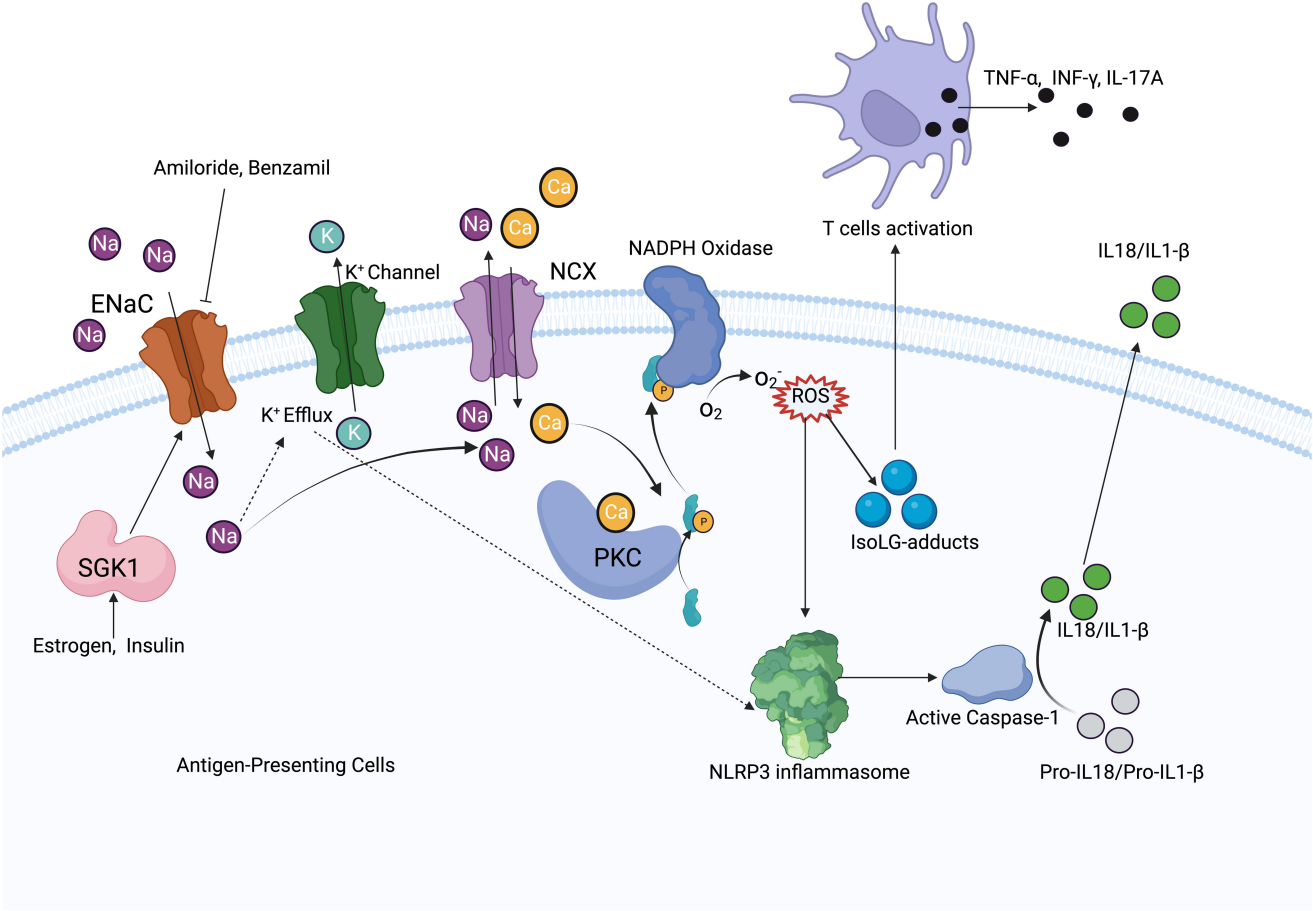
Mechanism diagram of ENaC activating immune system. In antigen-presenting cells, ENaC mediates Na^+^ influx, which increases Na^+^ exchange with Ca2^+^ via NCX. Elevated intracellular Ca2^+^ activates PKC and NADPH oxides to increase ROS production. ROS activates the NLRP3 inflammasome and promotes IsoLG-adducts formation, meanwhile, increased intracellular Na^+^ promotes K^+^ efflux, which also activates the NLRP3 inflammasome. NLRP3 inflammasome increases IL-1β, IL-18 production by activating caspase-1, IsoLG-adducts promotes T cells activation. ENaC expression is regulated by SGK1, while estrogen and insulin have regulatory effects on SGK1.

High salt exposure increases Na^+^ entry into cells through the ENaC, and increased intracellular Na^+^ acts as a driving force to promote NCX for Ca^2+^ exchange in antigen-presenting cells (APCs). Elevated intracellular Ca^2+^ increases ROS production and activates NLRP3 inflammasome, leading to T-cell activation and the release of inflammatory cytokines, which promotes Na^+^ reabsorption and hypertension (7). Increased Na^+^ leads to IsoLG-adduct formation and the expression of tumor necrosis factor (TNF)α, interleukin (IL)-6, and IL-1β. IsoLG-adducts as neoantigens can activate T cells (9), while the inhibition of NADPH-oxidase reduces monocyte activation and IsoLG-adduct formation (76) (**Figure 3**). It has been found that αENaC is overexpressed in neutrophils in hypertensive patients (77). SGK1 in APCs mediates high salt-induced expression, and the assembly of α and γENaC promotes the expression of IL-1β and the formation of IsoLG-adducts in salt-sensitive hypertension. Less endothelial dysfunction and blunted hypertension have been found both in SGK1 knockout mice and mice with application of SGK1 inhibitors (78).

A high-sodium diet (>150 mM) increases ENaC expression and promotes vascular endothelial cell stiffness and dysfunction (79). The ENaC is a key molecule of endothelial dysfunction in cardiovascular fibrosis and stiffening; activation of the ENaC on vascular endothelial cells increases oxidative stress and endothelial cell permeability, leading to impaired NO release and the formation of an inflammatory microenvironment (80). Aortic endothelium stiffness and dysfunction is reduced in αENaC knockout mice, including decreased endoplasmic reticulum stress and oxidative stress, and reduced endothelium permeability and expression of proinflammatory cytokines; endothelium NO synthase is also activated (81). Estrogen activates ENaC via SGK-1 in vascular endothelial cells, leading to a higher risk of arterial stiffening in women, which can be reduced by amiloride (82). Knockdown of αENaC in endothelial cells leads to a decrease of cortical stiffness; conversely, αENaC overexpression leads to an increase of cortical stiffness in vascular endothelial cells and promotes oxidative stress and inflammation in aortic tissues (83); this effect is related to AMP-activated protein kinase α (AMPKα) and sirtuin 1-mediated endothelial NO synthase (eNOS) activation (84). A high-fat diet leads to activation of ENaC-mediated inflammation and increases secretion of TNFα, IL-1β, IL-6, vascular cell adhesion molecule (VCAM)-1 and intracellular adhesion molecule (ICAM)-1, which in turn leads to endothelial dysfunction and vascular sclerosis. Benzamil is a specific inhibitor of ENaC that can reduce the inflammation induced by a high-fat diet (85).

The ENaC increases cardiac endothelium permeability, promotes macrophage recruitment and M1 polarization, and leads to ventricular fibrosis and remodeling in female mice. Amiloride can attenuate the impaired left ventricular initial filling rate and relaxation time (86). The ENaC causes endothelium-dependent relaxation impairment in mice aorta through the ROS/COX-2-mediated SGK-1/Nedd4-2 signaling pathway; blocking the ENaC facilitates attenuation of hyperhomocysteinemia-induced cardiovascular system disease (79).

### 3.2 Epithelial sodium channel-related inflammation in the respiratory system

**Figure 4:**
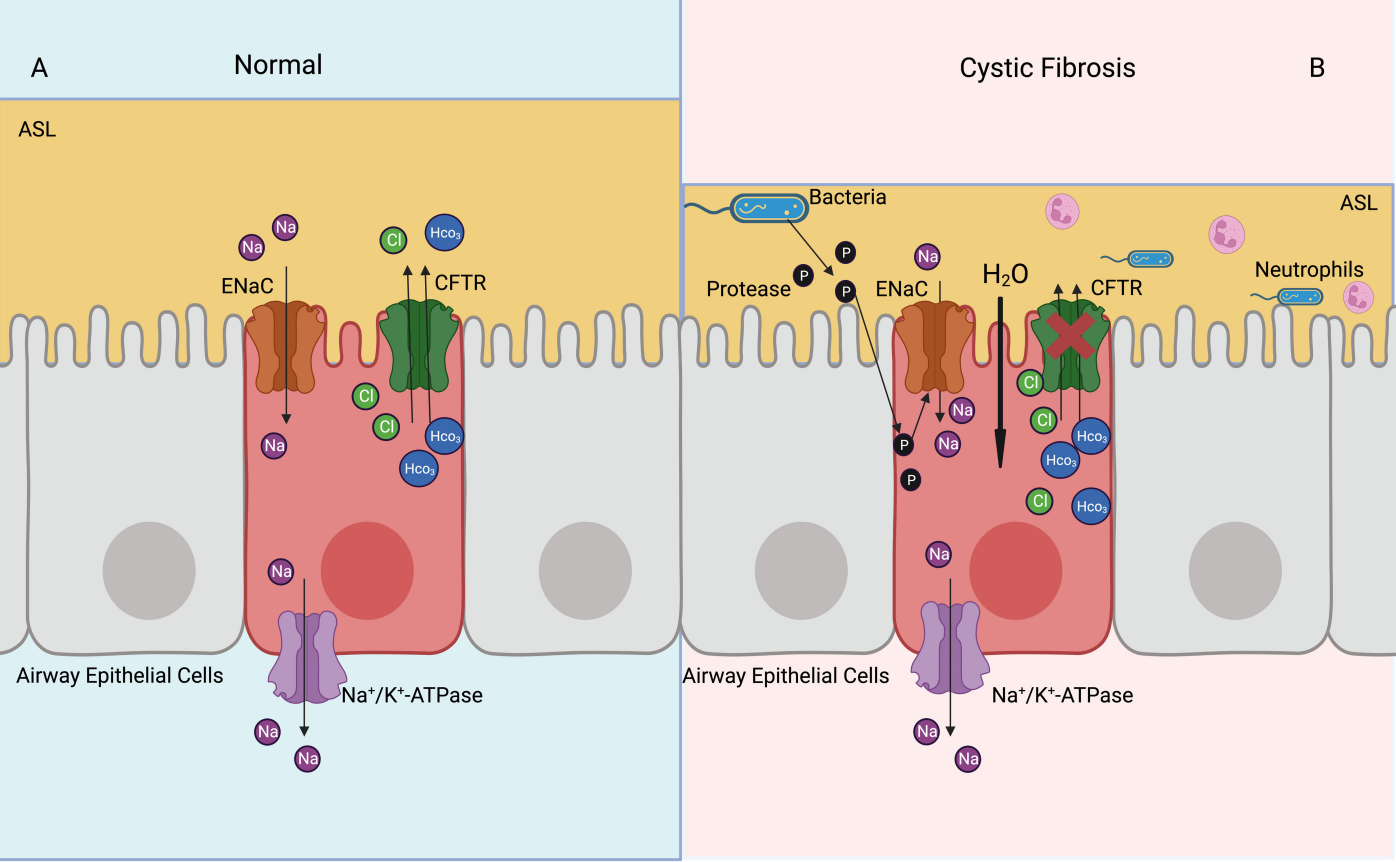
Activated ENaC exacerbates cystic fibrosis airway inflammation. **(A)** In the normal airway, ENaC mediates uptake of Na^+^ in the airway epithelial cells, CFTR mediates Cl- and HCO_3_- outflux, basolateral NKA expels Na^+^ out of cells, thus achieving a balance of intracellular ions transport and maintaining ASL normal function. **(B)** In the cystic fibrosis airway, CFTR dysfunction leads to airway bacterial infection and ASL dehydration. Proteases released by bacteria can activate ENaC, which increases Na^+^ into airway epithelial cells. Elevated intracellular osmotic pressure promotes moisture absorption, exacerbates ASL dehydration and airway inflammation.

Cystic fibrosis is a multisystem disease, characterized by mutations of the CFTR gene and repeated pulmonary infections (87). The ENaC has been shown to be overactive in cystic fibrosis, resulting in increased Na^+^ and water absorption from the airway lumen, eventually leading to mucus accumulation, bacterial infection, and airway inflammation (5) (**Figure 4**). The ENaC mediates Na^+^ influx and indirectly increases K^+^ efflux, which leads to NLRP3 inflammasome activation, further causing excessive IL-1β and IL-18 secretion (8). The bronchoalveolar lavage fluid has a higher number of inflammatory cytokines and chemokines, such as G-CSF, MCP-1, IL-5, and IL-6. In the βENaC overexpressed mice model, neutrophil extracellular traps were detected in the airways, even when there was no bacterial infection (88). Overexpression of βENaC increases the secretion of inflammatory cytokines and leads to conditions such as cystic fibrosis in mice; inhibition of the ENaC or NLRP3 inflammasome can improve the symptoms (8, 88) (**Figure 3**). βENaC transgenic mice developed chronic airway inflammation earlier than control group and had higher levels of CXC chemokines, MIP-2 and IL-13 (89). The pulmonary inflammation in mice overexpressing β and γENaC was increased, as evidenced by a significant increase in neutrophils, eosinophils, and lymphocytes (90). The application of antisense oligonucleotides to inhibit the expression of the ENaC in airway epithelial cells can reduce pulmonary inflammation (91). The ENaC is involved in pulmonary inflammation of muco-obstructive lung diseases (92) and acute respiratory distress syndrome (93); in similar molecular mechanisms, azithromycin improves obstructive lung diseases by targeting the ENaC (94).

Inflammatory cytokines can also modulate the ENaC, leading to increased pulmonary inflammation. IL-1α and IL-1β can induce αENaC expression through the NF-kB signaling pathway in mouse lung epithelial cells, where the extracellular signal-regulated kinase (ERK) and mitogen-activated protein kinase (MAPK) signaling pathway also plays a partial role (95). In small airway epithelial cells of mice injected intraperitoneally with high-mobility group box-1 protein (HMGB-1), the open probability of ENaC was increased, and the levels of IL-1β, IL-10, IL-6, IL-27, IL-17A and interferon (IFN)-β were significantly increased in the bronchoalveolar lavage fluid of these mice (96). Transforming growth factor (TGF)-β can mediate the internalization of βENaC through the TGF-β receptor 1 pathway in the alveolar epithelial cells, causing pulmonary edema in acute lung injury (97). TGF-β can inhibit the antioxidant system by internalization of the ENaC, activate the plasminogen activator inhibitor 1 (PAI-1) and NF-kB signaling pathway, and lead to inflammation and injury in the lung (98). ENaC activation can decrease ASL and increase inflammation in the airway. Resolvin D1 is a drug that can inhibit TNFα-mediated inflammation in macrophages; it also inhibits the decrease of ASL caused by ENaC activation while reducing IL-8 secretion by alveolar macrophages and enhancing the phagocytic capacity (99). Resolvin E1 regulates the expression of the ENaC and NKA through the PI3K/AKT/SGK1 signaling pathway that promotes alveolar fluid clearance and reduces inflammation in the lungs (100). T-helper cell type 2 (Th2)-dependent airway inflammation is related to reduced transcript levels of α, β, and γENaC (101).

Nedd4-2 inhibits the function of the ENaC by ubiquitinating lysine residues on the ENaC. Knockdown of Nedd4-2 ubiquitinates leads to increased ENaC activity and aseptic lung inflammation, which may be associated with fetal lethal lung disease (102). Upregulation of ENaC expression via the PI3K/Akt/Nedd4-2 signaling pathway can suppresse lipopolysaccharide-induced inflammation in acute lung injuries (103). Bacterial proteases contribute to increase ENaC activity, promote Na^+^ uptake by airway epithelial cells, decrease ASL and mucociliary clearance of airway, and exacerbate pulmonary inflammation (57, 104) (**Figure 4**).

### 3.3 Epithelial sodium channel-related inflammation in the kidneys

RAAS activates the ENaC in the collecting duct through increased reactive oxygen species (ROS); in particular, ROS raises PIP3 and decreases inhibition of the ENaC by arachidonic acid (105) (**Figure 2**). Renal inflammatory cytokines IL-1β, IL-6, TNFα, TGF-β, and collagen III were increased in βENaC knockout mice, and mean arterial blood pressure was elevated (106). When treated with a high salt intake, male db/db mice (a mouse model of obesity and diabetes) show higher renal fibrosis, albuminuria, and inflammatory cytokine expressions, including IL-1b, TNFa, IL-6, and IL-17A, than female mice, which is associated with ENaC dysregulation (107). IL-6 leads to elevated α, β, and γENaC in mouse cortical-collecting duct cells, which suggests that renal inflammation may lead to natriuresis via IL-6 (108). A high salt intake induces increased α and γENaC expression. Intracellular increased Na^+^ promotes IsoLG-adduct formation, leading to renal inflammation and hypertension; this process is SGK1 mediated. Inhibition of SGK1 in CD11c^+^ cells by knockdown or pharmacological inhibition can reduce nicotinamide adenine dinucleotide phosphate oxidase and ENaC expression, which plays a protective role against renal inflammation and hypertension (78, 109). The angiotensin-converting enzyme (ACE) has an important role in regulating Na^+^ absorption and diabetic renal inflammation. The ENaC expression is downregulated by 55% in ACE N-domain knockout diabetic mice compared with diabetic wild-type mice; IL-1β and TNFα are downregulated by 55% and 53%, respectively (110). TIP peptide can mimic the lectin-like domain of TNF and activate ENaC by binding α subunit. TIP peptide injected intraperitoneally into nephrotoxic serum nephritis mice reduces glomerular inflammation and proteinuria and decreases Th17 cell infiltration (111).

### 3.4 Epithelial sodium channel-related inflammation in the colon

IL-13 is increased in ulcerative colitis, which can inhibit the ENaC and SGK1 through the JAK1/2-STAT6-MAPK signaling pathways, and decreases Na^+^ reabsorption in the intestinal epithelium (112). Elevated proinflammatory cytokines such as TNFα and IFNγ inhibit β and γENaC expression; therefore, reducing colonic Na^+^ absorption leads to diarrhea in ulcerative colitis (113). In campylobacter jejuni-caused enteritis, β and γENaC dysfunction causes Na^+^ malabsorption, which leads to diarrhea and increased immune responses, and IFNγ, TNFα, IL-13, and IL-1β expressions are increased, which is confirmed by colonic biopsy (114). Campylobacter concisus downregulates β and γENaC through the IL-32-mediated ERK1/2 signaling pathway, impairs intestinal mucosal barrier function, and leads to inflammation and diarrhea (115). Aldosterone can upregulate γENaC expression through the MEK1/2 signaling pathway; TNFα, IFNγ, and IL-15 impair the promotion of aldosterone to γENaC in lymphocytic colitis, resulting in impaired Na^+^ absorption and diarrhea (116). The mechanism of reduced Na^+^ absorption in non-inflamed colon is impaired γENaC, which is similar to the mechanism of lymphocytic colitis (117).

### 3.5 Epithelial sodium channel-related inflammation in tumors

Chronic inflammation plays an important role in tumorigenesis, a high salt intake can lead to a microenvironment of chronic inflammation in tissues, and elevated Na^+^ in some tumor cells is associated with ENaC and ASIC overexpression (118). A high salt intake plays an important role in the transmembrane transport of glucose and glutamine, which helps to maintain the high active cellular state of tumor cells and promote tumor growth and metastasis (119). A high salt intake (0.15M NaCl) and IL-17 (0.1 nM) can upregulate γENaC, activate ROS, and reactive nitrogen (RNS), thus promoting the growth of breast cancer cells, and also promote inflammatory cytokine expression such as IL-6 and TNFα (120). However, some studies show that a high expression of αENaC inhibits breast cancer progression and migration, while a low expression of αENaC promotes the proliferation of breast cancer cells (121). The role of ENaC in tumor growth and metastasis has been extensively studied, but the role of ENaC-induced inflammation in tumors is lacking and needs further research.

### 3.6 Epithelial sodium channel-related inflammation in other areas

PHA is a multisystemic disease caused by ENaC mutations, manifesting as sweat gland duct occlusion and eccrine glands inflammation due to salt accumulation developing into miliaria rubra, folliculitis, and atopic dermatitis-like skin lesions (122). The barrier function disruption of skin epithelium leads to increased Na^+^ influx through ENaC, which activates fibroblasts via the COX-2/PGE2 pathway, resulting in fibrosis and the increased secretion of inflammatory cytokines. *In vivo* experiments verified that inhibition of ENaC or COX-2 significantly reduces scar formation (123). In the conditional βENaC meibomian gland knockout mouse model, inflammatory cell infiltration is significantly increased, inflammatory cytokine (IL-1β, IL-8, IL13, and Ym1) expression is significantly higher, and the incidence of other ocular surface diseases such as corneal opacification, ulceration, neovascularization is increased, which is one of the manifestations of PHA (124).

The ENaC is related to chronic rhinosinusitis, which manifests as significantly decreased α and βENaC mRNA levels in chronic rhinosinusitis patients (125). Lipopolysaccharide injected into the middle ear cavity can decrease ENaC expression and induce middle ear inflammation (126). Transtympanic injection of urban particulate matter leads to inflammatory cell infiltration and increased vascular space in the middle ear, along with decreased ENaC expression, which is associated with development of otitis (127). Inflammation can modulate ENaC-mediated Na^+^ uptake in taste buds, IL-1β induces an increase in Na^+^ transport, and, conversely, TNFα leads to a decrease in Na^+^ transport through the ENaC, which is related to the modulation of taste function during disease to limit Na^+^ consumption (128). Curcumin maintains tight junction proteins’ integrity, promotes ENaC and NKA expression, and decreases inflammation in hypoxia-induced cerebral edema, as evidenced by decreased NF-kB and inflammatory cytokines (IL-1, IL-2, IL-18, and TNFα) and an increase in anti-inflammatory cytokine (IL-10) expression. Migration of macrophages is important for phagocytosis of pathogens and cellular debris. ENaC promotes the migration and polarization of macrophages and amiloride reduces migration of macrophages by inhibiting the ENaC. Inflammatory cytokines IFNγ and TNFα can reduce the expression of αENaC and decrease the migration of macrophages (129).

## 4 Conclusions

The ENaC-mediated increase of intracellular Na^+^ can further promote Ca^2+^ influx and K^+^ efflux; intracellular Ca^2+^ overload activates downstream inflammatory signaling pathways, which is a key pathogenic mechanism of ENaC-related inflammation. There are several posttranslational modifications that have been reported to have a regulatory effect on the ENaC, but more research is still needed to demonstrate the regulatory role of other modifications. ENaC dysfunction disrupts intracellular ion homeostasis; the role of the ENaC on other ions and channels and the consequent changes in physiological function are not well studied. Regulation of ENaC expression by extracellular Na^+^ is reversed in the renal epithelium and vascular endothelium; the exact mechanism needs further investigation. The role of ENaC-related inflammation in tumor growth and migration needs further investigation.

## Author contributions

YC, XY, and ZY wrote and edited the manuscript. SZ, JZ, and WG designed and guided the study. All authors contributed to the article and approved the submitted version.

## References

[1] Kaulich E McCubbin PTN Schafer WR Walker DS. Physiological insight into the conserved properties of Caenorhabditis elegans acid-sensing degenerin/epithelial sodium channels. J Physiol (2022) 601(9):1625–53. doi: 10.1113/JP283238 PMC10424705 36200489

[2] Baldin JP Barth D Fronius M. Epithelial na(+) channel (ENaC) formed by one or two subunits forms functional channels that respond to shear force. Front Physiol (2020) 11:141. doi: 10.3389/fphys.2020.00141 32256376 PMC7090232

[3] Garcia-Rubio D Martinez-Vieyra I de la Mora MB Fuentes-Garcia MA Cerecedo D. Clinical application of epithelial sodium channel (ENaC) as a biomarker for arterial hypertension. Biosensors (Basel) (2022) 12(10):806. doi: 10.3390/bios12100806 36290943 PMC9599886

[4] Negussie AB Dell AC Davis BA Geibel JP. Colonic fluid and electrolyte transport 2022: an update. Cells (2022) 11(10):1712. doi: 10.3390/cells11101712 35626748 PMC9139964

[5] Blacona G Raso R Castellani S Pierandrei S Del Porto P Ferraguti G.?Downregulation of epithelial sodium channel (ENaC) activity in cystic fibrosis cells by epigenetic targeting. Cell Mol Life Sci (2022) 79(5):257. doi: 10.1007/s00018-022-04190-9 35462606 PMC9035428

[6] Persaud A Jiang C Liu Z Kefalas G Demian WL Rotin D. Elevated intracellular Na(+) and osmolarity stimulate catalytic activity of the ubiquitin ligase Nedd4-2. Proc Natl Acad Sci U.S.A. (2022) 119(30):e2122495119. doi: 10.1073/pnas.2122495119 35858421 PMC9335340

[7] Pitzer A Elijovich F Laffer CL Ertuglu LA Sahinoz M Saleem M. DC ENaC-dependent inflammasome activation contributes to salt-sensitive hypertension. Circ Res (2022) 131(4):328–44. doi: 10.1161/CIRCRESAHA.122.320818 PMC9357159 35862128

[8] Scambler T Jarosz-Griffiths HH Lara-Reyna S Pathak S Wong C Holbrook J. ENaC-mediated sodium influx exacerbates NLRP3-dependent inflammation in cystic fibrosis. Elife (2019) 8:e49248. doi: 10.7554/eLife.49248 31532390 PMC6764826

[9] Barbaro NR Foss JD Kryshtal DO Tsyba N Kumaresan S Xiao L. Dendritic cell amiloride-sensitive channels mediate sodium-induced inflammation and hypertension. Cell Rep (2017) 21(4):1009–20. doi: 10.1016/j.celrep.2017.10.002 PMC5674815 29069584

[10] Kaulich E Grundy LJ Schafer WR Walker DS. The diverse functions of the DEG/ENaC family: linking genetic and physiological insights. J Physiol (2022) 601( 9):1521–42. doi: 10.1113/JP283335 PMC10148893 36314992

[11] Artunc F Bohnert BN Schneider JC Staudner T Sure F Ilyaskin AV. Proteolytic activation of the epithelial sodium channel (ENaC) by factor VII activating protease (FSAP) and its relevance for sodium retention in nephrotic mice. Pflugers Arch (2022) 474(2):217–29. doi: 10.1007/s00424-021-02639-7 PMC8766372 34870751

[12] Kellenberger S Schild L. Epithelial sodium channel/degenerin family of ion channels: a variety of functions for a shared structure. Physiol Rev (2002) 82(3):735–67. doi: 10.1152/physrev.00007.2002 12087134

[13] Paudel P McDonald FJ Fronius M. The delta subunit of epithelial sodium channel in humans-a potential player in vascular physiology. Am J Physiol Heart Circ Physiol (2021) 320(2):H487–93. doi: 10.1152/ajpheart.00800.2020 33275523

[14] Gettings SM Maxeiner S Tzika M Cobain MRD Ruf I Benseler F. Two functional epithelial sodium channel isoforms are present in rodents despite pronounced evolutionary pseudogenization and exon fusion. Mol Biol Evol (2021) 38(12):5704–25. doi: 10.1093/molbev/msab271 PMC8662647 34491346

[15] Giraldez T Rojas P Jou J Flores C Alvarez de la Rosa D. The epithelial sodium channel delta-subunit: new notes for an old song. Am J Physiol Renal Physiol (2012) 303(3):F328–38. doi: 10.1152/ajprenal.00116.2012 22573384

[16] Rotin D Staub O. Function and regulation of the epithelial Na(+) channel ENaC. Compr Physiol (2021) 11(3):2017–45. doi: 10.1002/cphy.c200012 34061979

[17] Baconguis I Bohlen CJ Goehring A Julius D Gouaux E. X-ray structure of acid-sensing ion channel 1-snake toxin complex reveals open state of a Na(+)-selective channel. Cell (2014) 156(4):717–29. doi: 10.1016/j.cell.2014.01.011 PMC4190031 24507937

[18] Kleyman TR Kashlan OB Hughey RP. Epithelial Na(+) channel regulation by extracellular and intracellular factors. Annu Rev Physiol (2018) 80:263–81. doi: 10.1146/annurev-physiol-021317-121143 PMC5811403 29120692

[19] Zhang L Wang X Chen J Kleyman TR Sheng S. Accessibility of ENaC extracellular domain central core residues. J Biol Chem (2022) 298(5):101860. doi: 10.1016/j.jbc.2022.101860 35339489 PMC9052164

[20] Staub O Gautschi I Ishikawa T Breitschopf K Ciechanover A Schild L. Regulation of stability and function of the epithelial Na+ channel (ENaC) by ubiquitination. EMBO J (1997) 16(21):6325–36. doi: 10.1093/emboj/16.21.6325 PMC1170239 9351815

[21] Shimkets RA Warnock DG Bositis CM Nelson-Williams C Hansson JH Schambelan M. Liddle's syndrome: heritable human hypertension caused by mutations in the beta subunit of the epithelial sodium channel. Cell (1994) 79(3):407–14. doi: 10.1016/0092-8674(94)90250-x 7954808

[22] Pradervand S Barker PM Wang Q Ernst SA Beermann F Grubb BR. Salt restriction induces pseudohypoaldosteronism type 1 in mice expressing low levels of the beta-subunit of the amiloride-sensitive epithelial sodium channel. Proc Natl Acad Sci U.S.A. (1999) 96(4):1732–7. doi: 10.1073/pnas.96.4.1732 PMC15577 9990093

[23] Hamilton KL Eaton DC. Single-channel recordings from amiloride-sensitive epithelial sodium channel. Am J Physiol (1985) 249(3 Pt 1):C200–7. doi: 10.1152/ajpcell.1985.249.3.C200 2412449

[24] Ehret E Jager Y Sergi C Merillat AM Peyrollaz T Anand D. Kidney-specific CAP1/Prss8-deficient mice maintain ENaC-mediated sodium balance through an aldosterone independent pathway. Int J Mol Sci (2022) 23(12):6745. doi: 10.3390/ijms23126745 35743186 PMC9224322

[25] He M Zhou T Niu Y Feng W Gu X Xu W. The protease corin regulates electrolyte homeostasis in eccrine sweat glands. PloS Biol (2021) 19(2):e3001090. doi: 10.1371/journal.pbio.3001090 33591965 PMC7909636

[26] Duncan JW Granger JP Ryan MJ Drummond HA. Interleukin-17 Reduces betaENaC via MAPK Signaling in Vascular Smooth Muscle Cells. Int J Mol Sci (2020) 21(8):2953. doi: 10.3390/ijms21082953 32331392 PMC7215799

[27] Brown EF Mitaera T Fronius M. COVID-19 and liquid homeostasis in the lung-A perspective through the epithelial sodium channel (ENaC) lens. Cells (2022) 11(11):1801. doi: 10.3390/cells11111801 35681496 PMC9180030

[28] Frindt G Meyerson JR Satty A Scandura JM Palmer LG. Expression of ENaC subunits in epithelia. J Gen Physiol (2022) 154(10):e202213124. doi: 10.1085/jgp.202213124 35939271 PMC9387651

[29] Ozdener MH Mahavadi S Mummalaneni S Lyall V. Relationship between ENaC regulators and SARS-CoV-2 virus receptor (ACE2) expression in cultured adult human fungiform (HBO) taste cells. Nutrients (2022) 14(13):2703. doi: 10.3390/nu14132703 35807883 PMC9268489

[30] Shabbir W Topcagic N Aufy M Oz M. CRISPR/Cas9 mediated knock down of delta-ENaC blunted the TNF-induced activation of ENaC in A549 cells. Int J Mol Sci (2021) 22(4):1858. doi: 10.3390/ijms22041858 33673381 PMC7917654

[31] Charles RP Guitard M Leyvraz C Breiden B Haftek M Haftek-Terreau Z. Postnatal requirement of the epithelial sodium channel for maintenance of epidermal barrier function. J Biol Chem (2008) 283(5):2622–30. doi: 10.1074/jbc.M708829200 18039670

[32] Chifflet S Hernandez JA. The epithelial sodium channel and the processes of wound healing. BioMed Res Int (2016) 2016:5675047. doi: 10.1155/2016/5675047 27493961 PMC4963570

[33] Wichmann L Althaus M. Evolution of epithelial sodium channels: current concepts and hypotheses. Am J Physiol Regul Integr Comp Physiol (2020) 319(4):R387–400. doi: 10.1152/ajpregu.00144.2020 32783689

[34] Jiang C Kawabe H Rotin D. The ubiquitin ligase Nedd4L regulates the Na/K/2Cl co-transporter NKCC1/SLC12A2 in the colon. J Biol Chem (2017) 292(8):3137–45. doi: 10.1074/jbc.M116.770065 PMC5336150 28087701

[35] Chen J He J Luo J Zhong S. Association of alphaENaC p. Ala663Thr Gene Polymorphism With Sudden Sensorineural Hearing Loss. Front Genet (2021) 12:659517. doi: 10.3389/fgene.2021.659517 35024042 PMC8744410

[36] Mutchler SM Kleyman TR. New insights regarding epithelial Na+ channel regulation and its role in the kidney, immune system and vasculature. Curr Opin Nephrol Hypertens (2019) 28(2):113–9. doi: 10.1097/MNH.0000000000000479 PMC6349474 30585851

[37] Zhang J Yuan HK Chen S Zhang ZR. Detrimental or beneficial: Role of endothelial ENaC in vascular function. J Cell Physiol (2022) 237(1):29–48. doi: 10.1002/jcp.30505 34279047

[38] Eaton DC Helms MN Koval M Bao HF Jain L. The contribution of epithelial sodium channels to alveolar function in health and disease. Annu Rev Physiol (2009) 71:403–23. doi: 10.1146/annurev.physiol.010908.163250 18831683

[39] Lin J Gettings SM Talbi K Schreiber R Taggart MJ Preller M. Pharmacological inhibitors of the cystic fibrosis transmembrane conductance regulator exert off-target effects on epithelial cation channels. Pflugers Arch (2023) 475(2):167–79. doi: 10.1007/s00424-022-02758-9 PMC9849171 36205782

[40] Mall MA Button B Johannesson B Zhou Z Livraghi A Caldwell RA. Airway surface liquid volume regulation determines different airway phenotypes in liddle compared with betaENaC-overexpressing mice. J Biol Chem (2010) 285(35):26945–55. doi: 10.1074/jbc.M110.151803 PMC2930694 20566636

[41] Pitzer AL Van Beusecum JP Kleyman TR Kirabo A. ENaC in salt-sensitive hypertension: kidney and beyond. Curr Hypertens Rep (2020) 22(9):69. doi: 10.1007/s11906-020-01067-9 32852643 PMC7452925

[42] Yang H Tenorio Lopes L Barioni NO Roeske J Incognito AV Baker J. The molecular makeup of peripheral and central baroreceptors: stretching a role for Transient Receptor Potential (TRP), Epithelial Sodium Channel (ENaC), Acid Sensing Ion Channel (ASIC), and Piezo channels. Cardiovasc Res (2022) 118(15):3052–70. doi: 10.1093/cvr/cvab334 34734981

[43] Xu C Chen Y Ramkumar N Zou CJ Sigmund CD Yang T. Collecting duct renin regulates potassium homeostasis in mice. Acta Physiol (Oxf) (2023) 237(1):e13899. doi: 10.1111/apha.13899 36264268 PMC10754139

[44] Chapman K Holmes M Seckl J. 11beta-hydroxysteroid dehydrogenases: intracellular gate-keepers of tissue glucocorticoid action. Physiol Rev (2013) 93(3):1139–206. doi: 10.1152/physrev.00020.2012 PMC3962546 23899562

[45] Pearce D Manis AD Nesterov V Korbmacher C. Regulation of distal tubule sodium transport: mechanisms and roles in homeostasis and pathophysiology. Pflugers Arch (2022) 474(8):869–84. doi: 10.1007/s00424-022-02732-5 PMC9338908 35895103

[46] Blass G Klemens CA Brands MW Palygin O Staruschenko A. Postprandial effects on ENaC-mediated sodium absorption. Sci Rep (2019) 9(1):4296. doi: 10.1038/s41598-019-40639-x 30862903 PMC6414683

[47] Deng W Li CY Tong J He J Zhao Y Wang DX. Insulin ameliorates pulmonary edema through the upregulation of epithelial sodium channel via the PI3K/SGK1 pathway in mice with lipopolysaccharide−induced lung injury. Mol Med Rep (2019) 19(3):1665–77. doi: 10.3892/mmr.2019.9809 PMC6390057 30628684

[48] Stockand JD Mironova EV Xiang H Soares AG Contreras J McCormick JA. Chronic activation of vasopressin-2 receptors induces hypertension in Liddle mice by promoting Na(+) and water retention. Am J Physiol Renal Physiol (2022) 323(4):F468–78. doi: 10.1152/ajprenal.00384.2021 PMC9485005 35900342

[49] Ma HP Eaton DC. Acute regulation of epithelial sodium channel by anionic phospholipids. J Am Soc Nephrol (2005) 16(11):3182–7. doi: 10.1681/ASN.2005040434 16192420

[50] Alli AA Bao HF Alli AA Aldrugh Y Song JZ Ma HP. Phosphatidylinositol phosphate-dependent regulation of Xenopus ENaC by MARCKS protein. Am J Physiol Renal Physiol (2012) 303(6):F800–11. doi: 10.1152/ajprenal.00703.2011 PMC3468524 22791334

[51] Tuna KM Liu BC Yue Q Ghazi ZM Ma HP Eaton DC. Mal protein stabilizes luminal membrane PLC-beta3 and negatively regulates ENaC in mouse cortical collecting duct cells. Am J Physiol Renal Physiol (2019) 317(4):F986–95. doi: 10.1152/ajprenal.00446.2018 PMC6843038 31364376

[52] Zhang DD Duan XP Xiao Y Wu P Gao ZX Wang WH. Deletion of renal Nedd4-2 abolishes the effect of high sodium intake (HS) on Kir4.1, ENaC, and NCC and causes hypokalemia during high HS. Am J Physiol Renal Physiol (2021) 320(5):F883–96. doi: 10.1152/ajprenal.00555.2020 PMC8174810 33818128

[53] Pierandrei S Truglio G Ceci F Del Porto P Bruno SM Castellani S. DNA methylation patterns correlate with the expression of SCNN1A, SCNN1B, and SCNN1G (Epithelial sodium channel, ENaC) genes. Int J Mol Sci (2021) 22(7):3754. doi: 10.3390/ijms22073754 33916525 PMC8038451

[54] Krueger B Yang L Korbmacher C Rauh R. The phosphorylation site T613 in the beta-subunit of rat epithelial Na(+) channel (ENaC) modulates channel inhibition by Nedd4-2. Pflugers Arch (2018) 470(4):649–60. doi: 10.1007/s00424-018-2115-2 29397423

[55] Ruffieux-Daidie D Staub O. Intracellular ubiquitylation of the epithelial Na+ channel controls extracellular proteolytic channel activation via conformational change. J Biol Chem (2011) 286(4):2416–24. doi: 10.1074/jbc.M110.176156 PMC3024735 21084303

[56] Buck TM Brodsky JL. Epithelial sodium channel biogenesis and quality control in the early secretory pathway. Curr Opin Nephrol Hypertens (2018) 27(5):364–72. doi: 10.1097/MNH.0000000000000438 29916852

[57] Anand D Hummler E Rickman OJ. ENaC activation by proteases. Acta Physiol (Oxf) (2022) 235(1):e13811. doi: 10.1111/apha.13811 35276025 PMC9540061

[58] Saha B Leite-Dellova DCA Demko J Sorensen MV Takagi E Gleason CE. WNK1 is a chloride-stimulated scaffold that regulates mTORC2 activity and ion transport. J Cell Sci (2022) 135(23):jcs260313. doi: 10.1242/jcs.260313 36373794 PMC9789407

[59] Deng W Qi D Tang XM Deng XY He J Wang DX. The Wnk4/Spak pathway stimulates alveolar fluid clearance by upregulation of epithelial sodium channel in mice with lipopolysaccharide-induced acute respiratory distress syndrome. Shock (2022) 58(1):68–77. doi: 10.1097/SHK.0000000000001945 35670456 PMC9415224

[60] Soares AG Mironova E Archer CR Contreras J Stockand JD Abd El-Aziz TM. Cisplatin decreases ENaC activity contributing to renal salt wasting syndrome. Cancers (Basel) (2020) 12(8):2140. doi: 10.3390/cancers12082140 32752278 PMC7464492

[61] Fedosova NU Habeck M Nissen P. Structure and function of Na,K-ATPase-the sodium-potassium pump. Compr Physiol (2021) 12(1):2659–79. doi: 10.1002/cphy.c200018 34964112

[62] Yang L Xu Y Gravotta D Frindt G Weinstein AM Palmer LG. ENaC and ROMK channels in the connecting tubule regulate renal K+ secretion. J Gen Physiol (2021) 153(8):e202112902. doi: 10.1085/jgp.202112902 34143184 PMC8217949

[63] Nesterov V Bertog M Korbmacher C. High baseline ROMK activity in the mouse late distal convoluted and early connecting tubule probably contributes to aldosterone-independent K(+) secretion. Am J Physiol Renal Physiol (2022) 322(1):F42–54. doi: 10.1152/ajprenal.00252.2021 34843658

[64] Liu L Yamamoto A Yamaguchi M Taniguchi I Nomura N Nakakuki M. Bicarbonate transport of airway surface epithelia in luminally perfused mice bronchioles. J Physiol Sci (2022) 72(1):4. doi: 10.1186/s12576-022-00828-2 35196991 PMC10717372

[65] Wall SM Verlander JW Romero CA. The renal physiology of pendrin-positive intercalated cells. Physiol Rev (2020) 100(3):1119–47. doi: 10.1152/physrev.00011.2019 PMC7474261 32347156

[66] Jang H Park Y Jang J. Serum and glucocorticoid-regulated kinase 1: Structure, biological functions, and its inhibitors. Front Pharmacol (2022) 13:1036844. doi: 10.3389/fphar.2022.1036844 36457711 PMC9706101

[67] Barrett PQ Guagliardo NA Bayliss DA. Ion channel function and electrical excitability in the zona glomerulosa: A network perspective on aldosterone regulation. Annu Rev Physiol (2021) 83:451–75. doi: 10.1146/annurev-physiol-030220-113038 PMC7903429 33176563

[68] Pham TD Elengickal AJ Verlander JW Al-Qusairi L Chen C Abood DC. Pendrin-null mice develop severe hypokalemia following dietary Na(+) and K(+) restriction: role of ENaC. Am J Physiol Renal Physiol (2022) 322(5):F486–97. doi: 10.1152/ajprenal.00378.2021 PMC8977139 35224991

[69] Walter C Rafael C Lasaad S Baron S Salhi A Crambert G. H,K-ATPase type 2 regulates gestational extracellular compartment expansion and blood pressure in mice. Am J Physiol Regul Integr Comp Physiol (2020) 318(2):R320–8. doi: 10.1152/ajpregu.00067.2019 31913688

[70] Boscardin E Perrier R Sergi C Maillard MP Loffing J Loffing-Cueni D. Plasma potassium determines NCC abundance in adult kidney-specific gammaENaC knockout. J Am Soc Nephrol (2018) 29(3):977–90. doi: 10.1681/ASN.2017030345 PMC5827588 29371419

[71] Kamei S Fujikawa H Nohara H Ueno-Shuto K Maruta K Nakashima R. Zinc deficiency via a splice switch in zinc importer ZIP2/SLC39A2 causes cystic fibrosis-associated MUC5AC hypersecretion in airway epithelial cells. EBioMedicine (2018) 27:304–16. doi: 10.1016/j.ebiom.2017.12.025 PMC5828551 29289532

[72] Gumz ML Lynch IJ Greenlee MM Cain BD Wingo CS. The renal H+-K+-ATPases: physiology, regulation, and structure. Am J Physiol Renal Physiol (2010) 298(1):F12–21. doi: 10.1152/ajprenal.90723.2008 19640897 PMC2806118

[73] Hanssens LS Duchateau J Casimir GJ. CFTR protein: not just a chloride channel? Cells (2021) 10(11):2844. doi: 10.3390/cells10112844 34831067 PMC8616376

[74] Almughem FA Aldossary AM Tawfik EA Alomary MN Alharbi WS Alshahrani MY. Cystic fibrosis: overview of the current development trends and innovative therapeutic strategies. Pharmaceutics (2020) 12(7):616. doi: 10.3390/pharmaceutics12070616 32630625 PMC7407299

[75] Hu R McDonough AA Layton AT. Sex differences in solute transport along the nephrons: effects of Na(+) transport inhibition. Am J Physiol Renal Physiol (2020) 319(3):F487–505. doi: 10.1152/ajprenal.00240.2020 PMC7509281 32744084

[76] Ruggeri Barbaro N Van Beusecum J Xiao L do Carmo L Pitzer A Loperena R. Sodium activates human monocytes via the NADPH oxidase and isolevuglandin formation. Cardiovasc Res (2021) 117(5):1358–71. doi: 10.1093/cvr/cvaa207 PMC8064439 33038226

[77] Reus-Chavarria E Martinez-Vieyra I Salinas-Nolasco C Chavez-Pina AE Mendez-Mendez JV Lopez-Villegas EO. Enhanced expression of the Epithelial Sodium Channel in neutrophils from hypertensive patients. Biochim Biophys Acta Biomembr (2019) 1861(2):387–402. doi: 10.1016/j.bbamem.2018.11.003 30423324

[78] Van Beusecum JP Barbaro NR McDowell Z Aden LA Xiao L Pandey AK. High salt activates CD11c(+) antigen-presenting cells via SGK (Serum glucocorticoid kinase) 1 to promote renal inflammation and salt-sensitive hypertension. Hypertension (2019) 74(3):555–63. doi: 10.1161/HYPERTENSIONAHA.119.12761 PMC6687568 31280647

[79] Liang C Wang QS Yang X Zhu D Sun Y Niu N. Homocysteine causes endothelial dysfunction via inflammatory factor-mediated activation of epithelial sodium channel (ENaC). Front Cell Dev Biol (2021) 9:672335. doi: 10.3389/fcell.2021.672335 34222246 PMC8247579

[80] Hill MA Jaisser F Sowers JR. Role of the vascular endothelial sodium channel activation in the genesis of pathologically increased cardiovascular stiffness. Cardiovasc Res (2022) 118(1):130–40. doi: 10.1093/cvr/cvaa326 PMC8752352 33188592

[81] Jia G Habibi J Aroor AR Hill MA Yang Y Whaley-Connell A. Epithelial sodium channel in aldosterone-induced endothelium stiffness and aortic dysfunction. Hypertension (2018) 72(3):731–8. doi: 10.1161/HYPERTENSIONAHA.118.11339 PMC6202124 29987101

[82] Padilla J Woodford ML Lastra-Gonzalez G Martinez-Diaz V Fujie S Yang Y. Sexual dimorphism in obesity-associated endothelial ENaC activity and stiffening in mice. Endocrinology (2019) 160(12):2918–28. doi: 10.1210/en.2019-00483 PMC6853665 31617909

[83] Jeggle P Callies C Tarjus A Fassot C Fels J Oberleithner H. Epithelial sodium channel stiffens the vascular endothelium in *vitro* and in Liddle mice. Hypertension (2013) 61(5):1053–9. doi: 10.1161/HYPERTENSIONAHA.111.199455 23460285

[84] Sowers JR Habibi J Aroor AR Yang Y Lastra G Hill MA. Epithelial sodium channels in endothelial cells mediate diet-induced endothelium stiffness and impaired vascular relaxation in obese female mice. Metabolism (2019) 99:57–66. doi: 10.1016/j.metabol.2019.153946 31302199 PMC6901094

[85] Niu N Yang X Zhang BL Liang C Zhu D Wang QS. Endothelial epithelial sodium channel involves in high-fat diet-induced atherosclerosis in low-density lipoprotein receptor-deficient mice. Biochim Biophys Acta Mol Basis Dis (2021) 1867(1):165989. doi: 10.1016/j.bbadis.2020.165989 33065235

[86] Jia G Habibi J Aroor AR Hill MA DeMarco VG Lee LE. Enhanced endothelium epithelial sodium channel signaling prompts left ventricular diastolic dysfunction in obese female mice. Metabolism (2018) 78:69–79. doi: 10.1016/j.metabol.2017.08.008 28920862

[87] Mall MA. ENaC inhibition in cystic fibrosis: potential role in the new era of CFTR modulator therapies. Eur Respir J (2020) 56(6):2000946. doi: 10.1183/13993003.00946-2020 32732328 PMC7758539

[88] Tucker SL Sarr D Rada B. Neutrophil extracellular traps are present in the airways of ENaC-overexpressing mice with cystic fibrosis-like lung disease. BMC Immunol (2021) 22(1):7. doi: 10.1186/s12865-021-00397-w 33478382 PMC7819174

[89] Zhou-Suckow Z Duerr J Hagner M Agrawal R Mall MA. Airway mucus, inflammation and remodeling: emerging links in the pathogenesis of chronic lung diseases. Cell Tissue Res (2017) 367(3):537–50. doi: 10.1007/s00441-016-2562-z 28108847

[90] Livraghi-Butrico A Wilkinson KJ Volmer AS Gilmore RC Rogers TD Caldwell RA. Lung disease phenotypes caused by overexpression of combinations of alpha-, beta-, and gamma-subunits of the epithelial sodium channel in mouse airways. Am J Physiol Lung Cell Mol Physiol (2018) 314(2):L318–31. doi: 10.1152/ajplung.00382.2017 PMC5866504 29074490

[91] Crosby JR Zhao C Jiang C Bai D Katz M Greenlee S. Inhaled ENaC antisense oligonucleotide ameliorates cystic fibrosis-like lung disease in mice. J Cyst Fibros (2017) 16(6):671–80. doi: 10.1016/j.jcf.2017.05.003 28539224

[92] Carroll EL Bailo M Reihill JA Crilly A Lockhart JC Litherland GJ. Trypsin-like proteases and their role in muco-obstructive lung diseases. Int J Mol Sci (2021) 22(11):5817. doi: 10.3390/ijms22115817 34072295 PMC8199346

[93] Zhang L Gao J Qin C Liang Y Chen S Hei F. Inflammatory alveolar macrophage-derived microvesicles damage lung epithelial cells and induce lung injury. Immunol Lett (2022) 241:23–34. doi: 10.1016/j.imlet.2021.10.008 34740720

[94] Fujikawa H Kawakami T Nakashima R Nasu A Kamei S Nohara H. Azithromycin inhibits constitutive airway epithelial sodium channel activation in vitro and modulates downstream pathogenesis in vivo. Biol Pharm Bull (2020) 43(4):725–30. doi: 10.1248/bpb.b19-01091 32009028

[95] Mustafa SB Hernandez TF Johnson-Pais TL Kumar PA Petershack JA Henson BM. IL-1 promotes alpha-epithelial Sodium Channel (alpha-ENaC) expression in murine lung epithelial cells: involvement of NF-kappaB. J Cell Commun Signal (2020) 14(3):303–14. doi: 10.1007/s12079-019-00533-7 PMC7511495 31659629

[96] Grant GJ Liou TG Paine 3R Helms MN. High-mobility group box-1 increases epithelial sodium channel activity and inflammation via the receptor for advanced glycation end products. Am J Physiol Cell Physiol (2020) 318(3):C570–80. doi: 10.1152/ajpcell.00291.2019 PMC7099525 31913693

[97] Peters DM Vadasz I Wujak L Wygrecka M Olschewski A Becker C. TGF-beta directs trafficking of the epithelial sodium channel ENaC which has implications for ion and fluid transport in acute lung injury. Proc Natl Acad Sci U.S.A. (2014) 111(3):E374–83. doi: 10.1073/pnas.1306798111 PMC3903252 24324142

[98] Hamidi SH Kadamboor Veethil S Hamidi SH. Role of pirfenidone in TGF-beta pathways and other inflammatory pathways in acute respiratory syndrome coronavirus 2 (SARS-Cov-2) infection: a theoretical perspective. Pharmacol Rep (2021) 73(3):712–27. doi: 10.1007/s43440-021-00255-x PMC8057922 33880743

[99] Ringholz FC Higgins G Hatton A Sassi A Moukachar A Fustero-Torre C. Resolvin D1 regulates epithelial ion transport and inflammation in cystic fibrosis airways. J Cyst Fibros (2018) 17(5):607–15. doi: 10.1016/j.jcf.2017.11.017 29233471

[100] Luo J Zhang WY Li H Zhang PH Tian C Wu CH. Pro-resolving mediator resolvin E1 restores alveolar fluid clearance in acute respiratory distress syndrome. Shock (2022) 57(4):565–75. doi: 10.1097/SHK.0000000000001865 35271545

[101] Anagnostopoulou P Dai L Schatterny J Hirtz S Duerr J Mall MA. Allergic airway inflammation induces a pro-secretory epithelial ion transport phenotype in mice. Eur Respir J (2010) 36(6):1436–47. doi: 10.1183/09031936.00181209 20413543

[102] Leitz DHW Duerr J Mulugeta S Seyhan Agircan A Zimmermann S Kawabe H. Congenital deletion of nedd4-2 in lung epithelial cells causes progressive alveolitis and pulmonary fibrosis in neonatal mice. Int J Mol Sci (2021) 22(11):6146. doi: 10.3390/ijms22116146 34200296 PMC8201155

[103] Jiang Y Xia M Xu J Huang Q Dai Z Zhang X. Dexmedetomidine alleviates pulmonary edema through the epithelial sodium channel (ENaC) via the PI3K/Akt/Nedd4-2 pathway in LPS-induced acute lung injury. Immunol Res (2021) 69(2):162–75. doi: 10.1007/s12026-021-09176-6 PMC8106593 33641076

[104] Thibodeau PH Butterworth MB. Proteases, cystic fibrosis and the epithelial sodium channel (ENaC). Cell Tissue Res (2013) 351(2):309–23. doi: 10.1007/s00441-012-1439-z PMC5310219 22729487

[105] Gonzalez-Vicente A Hong N Garvin JL. Effects of reactive oxygen species on renal tubular transport. Am J Physiol Renal Physiol (2019) 317(2):F444–55. doi: 10.1152/ajprenal.00604.2018 PMC6732451 31215804

[106] Drummond HA Grifoni SC Abu-Zaid A Gousset M Chiposi R Barnard JM. Renal inflammation and elevated blood pressure in a mouse model of reduced beta-ENaC. Am J Physiol Renal Physiol (2011) 301(2):F443–9. doi: 10.1152/ajprenal.00694.2010 PMC3154591 21543417

[107] Veiras LC Shen JZY Bernstein EA Regis GC Cao D Okwan-Duodu D. Renal Inflammation Induces Salt Sensitivity in Male db/db Mice through Dysregulation of ENaC. J Am Soc Nephrol (2021) 32(5):1131–49. doi: 10.1681/ASN.2020081112 PMC8259671 33731332

[108] Li K Guo D Zhu H Hering-Smith KS Hamm LL Ouyang J. Interleukin-6 stimulates epithelial sodium channels in mouse cortical collecting duct cells. Am J Physiol Regul Integr Comp Physiol (2010) 299(2):R590–5. doi: 10.1152/ajpregu.00207.2009 PMC2928617 20504903

[109] Valinsky WC Touyz RM Shrier A. Aldosterone, SGK1, and ion channels in the kidney. Clin Sci (Lond) (2018) 132(2):173–83. doi: 10.1042/CS20171525 PMC5817097 29352074

[110] Eriguchi M Bernstein EA Veiras LC Khan Z Cao DY Fuchs S. The absence of the ACE N-domain decreases renal inflammation and facilitates sodium excretion during diabetic kidney disease. J Am Soc Nephrol (2018) 29(10):2546–61. doi: 10.1681/ASN.2018030323 PMC6171283 30185469

[111] Madaio MP Czikora I Kvirkvelia N McMenamin M Yue Q Liu T. The TNF-derived TIP peptide activates the epithelial sodium channel and ameliorates experimental nephrotoxic serum nephritis. Kidney Int (2019) 95(6):1359–72. doi: 10.1016/j.kint.2018.12.022 PMC6534471 30905471

[112] Dames P Bergann T Fromm A Bucker R Barmeyer C Krug SM. Interleukin-13 affects the epithelial sodium channel in the intestine by coordinated modulation of STAT6 and p38 MAPK activity. J Physiol (2015) 593(24):5269–82. doi: 10.1113/JP271156 PMC4704517 26365358

[113] Amasheh S Barmeyer C Koch CS Tavalali S Mankertz J Epple HJ. Cytokine-dependent transcriptional down-regulation of epithelial sodium channel in ulcerative colitis. Gastroenterology (2004) 126(7):1711–20. doi: 10.1053/j.gastro.2004.03.010 15188166

[114] Bucker R Krug SM Moos V Bojarski C Schweiger MR Kerick M. Campylobacter jejuni impairs sodium transport and epithelial barrier function via cytokine release in human colon. Mucosal Immunol (2018) 11(2):474–85. doi: 10.1038/mi.2017.66 28766554

[115] Nattramilarasu PK Bucker R Lobo de Sa FD Fromm A Nagel O Lee IM. Campylobacter concisus Impairs Sodium Absorption in Colonic Epithelium via ENaC Dysfunction and Claudin-8 Disruption. Int J Mol Sci (2020) 21(2):373. doi: 10.3390/ijms21020373 31936044 PMC7013563

[116] Barmeyer C Erko I Fromm A Bojarski C Loddenkemper C Dames P. ENaC dysregulation through activation of MEK1/2 contributes to impaired Na+ Absorption in lymphocytic colitis. Inflammation Bowel Dis (2016) 22(3):539–47. doi: 10.1097/MIB.0000000000000646 26658215

[117] Zeissig S Bergann T Fromm A Bojarski C Heller F Guenther U. Altered ENaC expression leads to impaired sodium absorption in the noninflamed intestine in Crohn's disease. Gastroenterology (2008) 134(5):1436–47. doi: 10.1053/j.gastro.2008.02.030 18355814

[118] Chen J Liu X Huang H Zhang F Lu Y Hu H. High salt diet may promote progression of breast tumor through eliciting immune response. Int Immunopharmacol (2020) 87:106816. doi: 10.1016/j.intimp.2020.106816 32721893

[119] Leslie TK Brackenbury WJ. Sodium channels and the ionic microenvironment of breast tumours. J Physiol (2022) 601(9):1543–53. doi: 10.1113/JP282306 PMC10953337 36183245

[120] Amara S Ivy MT Myles EL Tiriveedhi V. Sodium channel gammaENaC mediates IL-17 synergized high salt induced inflammatory stress in breast cancer cells. Cell Immunol (2016) 302:1–10. doi: 10.1016/j.cellimm.2015.12.007 26723502 PMC4792675

[121] Ware AW Harris JJ Slatter TL Cunliffe HE McDonald FJ. The epithelial sodium channel has a role in breast cancer cell proliferation. Breast Cancer Res Treat (2021) 187(1):31–43. doi: 10.1007/s10549-021-06133-7 33630195

[122] Shabbir W Topcagic N Aufy M. Activation of autosomal recessive Pseudohypoaldosteronism1 ENaC with aldosterone. Eur J Pharmacol (2021) 901:174090. doi: 10.1016/j.ejphar.2021.174090 33831414

[123] Xu W Hong SJ Zeitchek M Cooper G Jia S Xie P. Hydration status regulates sodium flux and inflammatory pathways through epithelial sodium channel (ENaC) in the skin. J Invest Dermatol (2015) 135(3):796–806. doi: 10.1038/jid.2014.477 25371970

[124] Sarada SK Titto M Himadri P Saumya S Vijayalakshmi V. Curcumin prophylaxis mitigates the incidence of hypobaric hypoxia-induced altered ion channels expression and impaired tight junction proteins integrity in rat brain. J Neuroinflamm (2015) 12:113. doi: 10.1186/s12974-015-0326-4 PMC4464871 26048285

[125] Saber A Nakka SS Hussain R Hugosson S. Staphylococcus aureus in chronic rhinosinusitis: the effect on the epithelial chloride channel (cystic fibrosis transmembrane conductance regulator, CFTR) and the epithelial sodium channel (ENaC) physiology. Acta Otolaryngol (2019) 139(7):652–8. doi: 10.1080/00016489.2019.1603513 31050570

[126] Song JJ Kwon SK Cho CG Park SW Chae SW. Expression of ENaC in LPS-induced inflammation of middle ear mucosa. Acta Otolaryngol (2012) 132(11):1145–50. doi: 10.3109/00016489.2012.697640 22830999

[127] Park MK Chae SW Kim HB Cho JG Song JJ. Middle ear inflammation of rat induced by urban particles. Int J Pediatr Otorhinolaryngol (2014) 78(12):2193–7. doi: 10.1016/j.ijporl.2014.10.011 25458159

[128] Kumarhia D He L McCluskey LP. Inflammatory stimuli acutely modulate peripheral taste function. J Neurophysiol (2016) 115(6):2964–75. doi: 10.1152/jn.01104.2015 PMC4922616 27009163

[129] Nemeth Z Hildebrandt E Parsa N Fleming AB Wasson R Pittman K. Epithelial sodium channels in macrophage migration and polarization: role of proinflammatory cytokines TNFalpha and IFNgamma. Am J Physiol Regul Integr Comp Physiol (2022) 323(5):R763–75. doi: 10.1152/ajpregu.00207.2022 PMC9639769 36189990

